# Isolated Knee Arthritis as Early and Only Symptom of Whipple's Disease

**DOI:** 10.1155/2018/3417934

**Published:** 2018-05-27

**Authors:** Dario Giunchi, Natalie Marcoli, Luca Deabate, Marco Delcogliano, Enrique Testa, Christian Candrian, Paolo Gaffurini

**Affiliations:** ^1^Servizio di Chirurgia e Ortopedia, Ospedale Regionale di Lugano, EOC, Ticino, Switzerland; ^2^Servizio di Reumatologia, Ospedale Regionale di Lugano, EOC, Ticino, Switzerland

## Abstract

We report a case of isolated Whipple's disease involving the knee of a 64-year-old female patient who presented recurrent monoarthritis whose origin was not clear. Initially, the cause of the gradually invalidating symptoms was related to a meniscal lesion and a diffuse minor grade chondropathy, but pain and functional impairment suggested that more exams were needed. Biopsies were performed during arthroscopy. The histology showed highly inflammatory infiltrates with PAS staining negative for *Tropheryma* while PCR revealed the infection with *Tropheryma whipplei*. This, following the recommendation of a rheumatologist and infectious disease specialist, led to biopsies of the gastrointestinal tract and analysis of the cerebrospinal fluid that showed no other organ involvement. This confirms the scientific literature that an isolated monoarthritis without involvement of the gastrointestinal tract caused by this bacterium is rare but can occur as an early manifestation of potentially fatal systemic disease. Moreover, a review of the scientific literature showed the uncertainty about epidemiology of this rare disease, suggesting that more and specific data are required.

## 1. Introduction

Whipple's disease is an extremely rare condition caused by a Gram-positive actinobacteria (*Tropheryma whipplei*), first described by George Hoyt Whipple in 1907 [1]. Classic Whipple's disease is a systemic infection characterized by arthralgias, fever, diarrhoea, malabsorption, and weight loss. Rarer still is an uncommon onset characterized by an isolated recurring monoarthritis. Since its first description in 1907, fewer than 1000 cases have been reported and just one another patient is known in the literature to have had the same onset of Whipple's disease (WD) [2, 3]. The disease affects mainly middle-aged men with a male-to-female ratio of 8 : 1 [4]. The prevalence rate is not known, but an estimated 1 case per million has been reported [4]. A more recent paper from Biagi et al. [5] reports an estimated prevalence in northwestern Italy of 3 per million (95% confidence interval). The annual incidence rate originally reported by Dobbins [6] was approximately 12 new cases per year worldwide. This low rate is related to pre-PCR testing, so the real incidence may be higher than this [3].

## 2. Case Presentation

A 64-year-old female patient presented herself at the orthopedic outpatient service of Ospedale Regionale di Lugano (ORL) for the first time in September 2016 after being seen by her rheumatologist. She started having left knee pain 4 years earlier presumably after a skiing accident. She then started having recurring knee pain with swelling, which was treated with anti-inflammatory drugs. The pain gradually became more intense and less responsive to her pain medications which prompted her to see a rheumatologist. Her attending rheumatologist found clinical signs of arthritis. Synovial fluid analysis then showed a highly inflammatory effusion with >10,000 cells/*μ*l with predominantly mononuclear cells, no crystals were identified, and culture was negative for bacterial growth. Family history, review of systems, and laboratory workup (rheumatoid factor, anticyclic citrullinated peptides (CCP), and antinuclear antibodies (ANA IF Hep-2)) showed no clear cause of the monoarthritis. She was then sent to our outpatient clinic for clinical evaluation and diagnostic arthroscopy.

In 2007, she was diagnosed with breast carcinoma and underwent left quadrantectomy followed by radiotherapy in 2008. No other preexisting conditions were known. She was not taking any medications regularly. Our patient has never been treated with corticosteroids or anti-TNF biologicals, nor she never had chemotherapy for her breast carcinoma. She had surgery and just radiotherapy 10 years before.

The clinical examination showed an afebrile patient in good general condition. Walking was possible for short distances, and only when aided by crutches, the left knee was warm, swollen, and painful on palpation accompanied by reduced passive and active ROM partly due to pain, and the rest of the clinical examination was within normal.

An MRI of the knee showed a grade II patellar chondropathy, a grade IV chondropathy of the femoral trochlea, and a complex lesion of the posterior horn of the medial meniscus and diffuse reactive synovitis.

Routine laboratory tests performed before surgery were within normal limits.

As planned, the arthroscopy was conducted and consisted of partial meniscectomy, synovialectomy, and biopsies. Due to the appearance of an intra-articular hematoma, a second arthroscopy was done five days later for lavage.

The histology showed chronic lymphofollicular synovitis rich in plasma cells. Moreover, hemosiderin depositions were reported. No PAS-positive inclusion bodies were found. The intuition of looking for *T. whipplei* came from the rheumatologist specialist during her consultation. The history of recurrent monoarthritis, inflammatory characteristic of the joint fluid, and the synovial biopsy showing chronic lymphoplasmacytic infiltrates were taken into consideration. The search for *T. whipplei* genome through PCR of the biopsy resulted positive. The results of real-time PCR have been confirmed by a synthetic plasmid of the laboratory itself. Moreover, the patient had a second opinion by another specialist who repeated the PCR of the synovial fluid which resulted positive, thus confirming our diagnosis. To exclude a systemic infection, an esophagogastroduodenoscopy was done six weeks after surgery as well as a lumbar puncture. No microscopic or macroscopic alteration was found at the biopsies of the superior gastrointestinal tract. Duodenal biopsy/PCR and CSF PCR for *Tropheryma whipplei* gave negative results following the same methodology previously mentioned. The serology for Borrelia, crystals, and culture was negative.

Two months after, follow-up clinical control was favorable showing full recovery of activities of daily living and pain-free ROM in both flexion and extension. No signs of inflammation were shown. The attending infectiologist prescribed empiric antibiotic therapy with ceftriaxone and co-trimoxazole.

Currently, 14 months after the first and last clinical consultation, the patient has a good life quality with no recurrence of the knee arthritis claiming just some light soreness after long walks which, in our opinion, is more of a mechanical problem than inflammatory.

## 3. Discussion

Whipple's disease is a rare bacterial infection whose transmission has been unclear. With the advent of 16S RNA gene amplification, *T. whipplei* has been isolated in water, and thus, an orofecal route and human-to-human transmission have been postulated [7, 8]. Upon contact with *Tropheryma whipplei*, the majority of cases will develop a protective immune response [9]. The existence of asymptomatic healthy carriers with *T. whipplei* isolated in the saliva and stool suggests that to develop a disease, there must be an underlying genetic predisposition causing an underlying immune defect [9]. Studies have shown that a TH1 immune defect causes a decrease in macrophage and T lymphocyte activity as well as the interaction in eliminating intracellulare *Tropheryma whipplei* [7, 10]. This immune defect confers a lifelong susceptibility to *Tropheryma whipplei* [9].

Joint involvement is frequent in Whipple's disease accounting for up to 87% [11] or 90% of cases [3]. Very few data are reported, but in those specific cases, symptoms related to gastrointestinal involvement follow the joint symptoms (arthralgias and seronegative arthritis) by a variable number of years ranging from several months as reported by Marth [11] to several decades [12]. The musculoskeletal manifestation can range from oligo-/polyarthritis, spondylodiscitis, and even erosive destructive polyarthritis [12, 13].

Several cases of isolated arthritis possibly due to *Tropheryma whipplei* have been reported; diagnosis was made through PCR of both synovial biopsy and synovial fluid without other organ involvement [2, 14].

We think that the clinical improvement was due to both surgical therapies and the antibiotics. In surgical terms, probably, the synovectomy has been more effective than the meniscectomy because of the severe synovial inflammatory reaction seen during the arthroscopy. It is not possible to quantify exactly which one had the greatest effect, but it is reasonable to expect that it is a combination of the three.

About surgical approaches in WD monoarthritis, there is no literature about this. We can state that, during a partial meniscectomy, a concurrent synovectomy would have been performed even if she had no infection because the inflammatory synovial reaction is well known to give pain, soreness, stiffness, and longer recovery to patients.

The choice of the antibiotics therapy for this case, ceftriaxone 2 g IV 14 days followed by co-trimoxazole 960 mg twice a day, has been taken by the infection disease specialist. This therapy, together with the diagnosis and the PCR results, has been confirmed by a second independent specialist in Zurich who prescribed the same Ab. Moreover, Marth [11] in 2016 proposed the same therapy as a standard treatment summarising other recent studies. Biagi et al. reports that there is no consensus about which one is the best therapy for WD, but standard therapy (ceftriaxone TMP-SMZ) for the first year(s) and then lifelong doxycycline seems to be a reasonable option [5, 15]. Dolmans et al. [16] reviewing the recent literature reported an alternative approach involving “doxycycline (200 mg/day) and hydroxychloroquine (600 mg/day) for 12 months and for localized *T. whipplei* infection, treatment with doxycycline (200 mg/day) and hydroxychloroquine (600 mg/day) for 12 to 18 months.” In our case, both the specialists consulted and agreed for the first mentioned therapy.

Referring to immunosuppressive therapies that can alter or induce a false diagnosis of WD [11, 16], our patient has never had those kind of treatments for her breast carcinoma nor for the more recent knee pain she suffered before WD diagnosis was made. As reported by T. Marth, immunosuppressive drugs especially tumour necrosis factor inhibitor (TNFI) may influence the clinical course of *T. whipplei* infection [9, 11] leading to WD. In our case, just common painkillers and nonsteroidal anti-inflammatory drugs were prescribed to her, but not corticosteroids.

The difficulty and delay of the diagnosis of Whipple's disease is caused in part by the variety of clinical presentations and its rarity. Diagnosis is confirmed by positive PAS staining, and the PCR as routine culture is not available. Up to 15% of cases are isolated or focal Whipple's disease (joint, CNS, or endocarditis) wherein no gastrointestinal manifestation is reported, and jejunal endoscopic findings and biopsies are normal [2, 3, 7]. The PCR is of importance especially in isolated chronic disease where a histologic PAS staining is difficult or might be negative [10]. Puechal suggested a diagnostic algorithm for Whipple's disease, highlighting the importance of both histologic and molecular diagnostic approach in the diagnosis of Whipple's disease [17].

Müller et al. [18] in 2005 stated that the PCR had 84% of sensitivity and 94% of specificity in diagnosing WD in the gastrointestinal tract but 100% of sensitivity in other organs. Specificity was not available. We are confident in our results thanks to the repeated tests, as we presented previously, and also by a second series of tests repeated on another sample collected by an independent specialist and analyzed in a second laboratory.

As in our case, repeated duodenal biopsy and PCR for *Tropheryma whipplei* resulted negative while the synovial biopsy showed positive PCR. The diagnosis of a probable isolated monoarthritis due to *Tropheryma whipplei* was given, and due to a potentially fatal course if left untreated, an empirical antibiotic therapy with ceftriaxone and co-trimoxazole to eradicate the bacillus was proposed. In case of recurring joint pains or inflammation of unknown or unclear etiology, *Tropheryma whipplei* infection remains a differential diagnosis to consider. A multidisciplinary management between the orthopedics and the rheumatologist led to an early diagnosis and treatment.

## 4. Conclusion

Whipple's disease is a rare systemic bacterial infection. Focal infections especially of the CNS, joints, and endocarditis can occur even in the absence of gastrointestinal involvement. The rarity of the disease, the varied clinical manifestation, and the difficulty in diagnosis can lead to potentially fatal outcomes if left untreated. A high index of suspicion in case of recurring joint inflammation of unknown or unclear etiology should prompt further diagnostic exams and procedure. A multidisciplinary management between the orthopedics and the rheumatologist in this case led to an early diagnosis and treatment.
